# Do Cortisol Levels Play a Role in Suicidal Behaviors and Non-Suicidal Self-Injuries in Children and Adolescents?—A Narrative Review

**DOI:** 10.3390/brainsci15030287

**Published:** 2025-03-08

**Authors:** Bartłomiej Sporniak, Monika Szewczuk-Bogusławska

**Affiliations:** Department of Psychiatry, Wroclaw Medical University, 50-367 Wroclaw, Poland; monika.szewczuk-boguslawska@umw.edu.pl

**Keywords:** cortisol, suicide, self-harm, adolescence

## Abstract

**Background/Objectives:** Suicidal behaviors (SBs) and non-suicidal self-injury (NSSI) are significant mental health concerns in children and adolescents. The hypothalamic–pituitary–adrenal (HPA) axis, of which cortisol is a key hormone, has been implicated in these behaviors. This narrative review aims to explore whether cortisol levels play a role in SBs and NSSI in youth and to synthesize current evidence on this topic. **Methods:** A comprehensive literature search was conducted on studies published through November 2024, using PubMed, Web of Science, and Google Scholar databases. Studies were screened for eligibility, including only human studies published in English, with no animal models or studies excluding cortisol levels. A narrative synthesis approach was used due to the methodological diversity across studies. Due to limited adolescent-focused research, studies involving adults were also considered. **Results:** Findings indicate inconsistent cortisol patterns in relation to SBs and NSSI. Elevated cortisol levels are linked to SBs, with some studies suggesting they may predict future suicide attempts, though no definitive cause-and-effect relationship is established. Conversely, cortisol levels in relation to NSSI show mixed results, with some studies reporting no differences. Cortisol responses to stress, measured by saliva, blood, and hair, reveal complex interactions with psychological factors such as depression and impulsivity, influencing cortisol secretion. **Discussion:** Despite some evidence pointing to a role of cortisol dysregulation in SBs and NSSI, the relationship remains unclear due to study heterogeneity, including small sample sizes and methodological variations. Gender and the type of stressor used in studies also complicate the findings. Future research should prioritize longitudinal studies, better control for confounding factors, and utilize more diverse cortisol assessment methods to clarify these links. **Conclusions:** While cortisol may play a role in the pathophysiology of SBs and NSSI, further research is needed to establish clearer, more reliable patterns. Identifying alterations in cortisol levels may aid in early detection and targeted interventions for at-risk adolescents.

## 1. Introduction

Non-suicidal self-injury (NSSI) and suicidal behaviors (SBs) in children and adolescents are critical global mental health issues, with NSSI often serving as a predictor of SBs [1]. Both behaviors may occur in the context of various psychiatric disorders or independently, but are frequently influenced by similar underlying factors.

One of the key risk factors for both NSSI and SBs is exposure to stress, including both past stressors (e.g., adverse childhood experiences (ACEs)) and current stressors (e.g., bullying and negative life events) [2,3]. Given the increasing recognition of stress in the mental health of young individuals, there has been growing attention on the role of the hypothalamic–pituitary–adrenal (HPA) axis, cortisol levels, and their relationship to mental health outcomes in adolescents. This leads to the central research question of this review: Do cortisol levels, as a marker of stress response, contribute to the onset, progression, and persistence of SBs and NSSI in adolescents?

Cortisol, a glucocorticoid produced by the adrenal glands, plays a central role in the body’s physiological stress response. The HPA axis regulates both immediate and long-term stress responses, influencing behaviors and neurobiological changes. Under stress, the hypothalamus releases corticotropin-releasing hormone (CRH), which triggers the release of adrenocorticotropic hormone (ACTH) from the pituitary gland, prompting cortisol production in the adrenal cortex [4]. Acting primarily through glucocorticoid receptors in the hippocampus, prefrontal cortex, and amygdala, cortisol influences neural plasticity, learning, memory, and stress recovery [5,6]. Chronic cortisol exposure can disrupt neurotransmitter systems, including serotonin, dopamine, and norepinephrine [7]. This dysregulation may also contribute to neuroinflammation [8], which has been linked to psychiatric disorders like depression and psychosis [9,10,11].

Dysregulation of the HPA axis and impaired cortisol secretion are thought to contribute to a range of mental health issues, including anxiety, depression, and psychosis [12,13,14,15,16]. However, not everyone exposed to stress develops mental disorders, suggesting that individual vulnerabilities, including HPA axis dysfunction, play a crucial role in the development of these conditions [17]. Stress is a critical factor in the onset and course of SBs and NSSI, with acute stress, such as psychiatric disorders or traumatic events, interacting with inherent vulnerability to shape neurobiological changes that may lead to these behaviors [18,19].

This review examines studies that investigate various cortisol measurements, including unstimulated, stimulated, and hair cortisol levels, to better understand the potential mechanisms linking cortisol dysregulation to NSSI and SBs in adolescents.

## 2. Materials and Methods

### 2.1. Study Design

Given the methodological heterogeneity across studies, a narrative review was chosen to allow for the integration of diverse findings and to present a comprehensive understanding of the relationship between cortisol and SBs and NSSI in children and adolescents [20]. The narrative synthesis approach was preferred to account for variations in study design, populations, methods of cortisol assessment, and outcome measures. This approach provides a broader overview of the existing literature and enables the identification of patterns or themes across studies. The methodology along with the search strategy is described in Supplementary Material.

### 2.2. Literature Search Strategy

A review of the electronic literature was conducted via the PubMed, Web of Science, and Google Scholar databases using a combination of the following terms: “cortisol” or “glucocorticoid” or “hpa axis” and “suicid*” or “non-suicidal self-injury” or “nssi” or “self-injury” or “self-harm” and “adolescence” or “adolescent” or “teen*” or “underage” or “children”.

The search was limited to articles published up to November 2024. Studies conducted on animal models were excluded, as they are not directly relevant to the human context of the review. Articles were also excluded if they did not report cortisol concentrations or focus on SBs or NSSI. A manual search of reference lists from included studies was also conducted to ensure that no relevant publications were overlooked. The inclusion of studies involving both adolescent and adult populations was aimed at broadening the scope of the findings due to the limited number of adolescent-focused studies. Due to resource constraints, the scope of this review was restricted to articles published in English, as the inclusion of articles in multiple languages would have required additional resources for translation and interpretation, potentially impacting the consistency and quality of the review process.

### 2.3. Study Selection and Screening

After the removal of duplicates, studies were initially screened based on titles and abstracts to assess their relevance. The screening was performed independently by two authors. Full-text articles were retrieved and assessed for eligibility. Any discrepancies between the authors were resolved through discussion and consensus. Studies that met the inclusion criteria were included in the synthesis.

The inclusion criteria for this review consisted of human studies that examined cortisol concentrations in relation to SBs or NSSI in adolescents or adults. These studies needed to involve male and female participants diagnosed with psychiatric disorders, excluding somatic diseases, neurological disorders, intellectual disabilities, and autism. Only articles published in English were considered for inclusion due to resource constraints. The exclusion criteria comprised studies conducted in animal models, studies that did not report cortisol levels or that did not focus on suicidality or NSSI, and articles published in languages other than English.

For data extraction and synthesis, key information was extracted from each study, including the study design (e.g., cohort, cross-sectional, longitudinal), sample characteristics (such as age, gender, and diagnostic criteria), methods of cortisol assessment (e.g., blood, saliva, hair), findings related to cortisol levels and their relationship with suicidality or NSSI, and any noted limitations of the study. Due to the substantial methodological diversity among the studies, a narrative synthesis was employed to integrate the findings. This approach allows for a comprehensive and context-sensitive analysis. The synthesis focused on identifying common themes, methodological trends, and differences across studies. Critical appraisal tools (such as the Newcastle–Ottawa Scale) were used to assess the quality and risk of bias of included studies, ensuring the reliability and validity of the findings presented.

### 2.4. Statistical Considerations

Given the varied study designs and outcome measures, statistical meta-analysis was not conducted. Instead, a descriptive summary of findings was provided. Where possible, effect sizes were noted to illustrate the magnitude of differences observed between groups. The review aimed to present the findings in a transparent and reproducible manner, providing clarity on the methodological approaches employed and the limitations of the evidence base.

### 2.5. Risk of Bias and Quality Appraisal

To enhance transparency and assess the robustness of the findings, each study’s methodological quality was appraised using appropriate quality appraisal tools (Newcastle–Ottawa Scale (NOS) for cohort and case-control studies). The NOS is a widely used tool for evaluating the methodological quality of non-randomized studies, including cohort and case-control studies. The NOS assigns a score based on three key domains: Selection, which evaluates the adequacy of participant selection, including the representativeness of the study groups and non-response rates; Comparability, which assesses how well the study groups are comparable, considering factors such as confounding variables and matching strategies; and Exposure, which evaluates how the exposure of interest (e.g., cortisol measurement, stress response) is assessed and whether the method used is reliable and valid. Each domain is assigned a score, and studies are awarded a maximum of 4 points for Selection, 2 points for Comparability, and 3 points for Exposure, for a total possible score of 9 points. Studies with scores of 6 or higher are considered to have acceptable methodological quality.

Studies with high risk of bias or methodological limitations were identified and discussed to mitigate their impact on the synthesis. Additionally, any potential conflicts of interest were noted.

## 3. Results

### 3.1. Unstimulated (Baseline) Cortisol Concentrations

The characteristics of the studies included in this narrative review are presented in Table 1. Cortisol levels are commonly assessed using various types of biological material, each providing unique insights into cortisol dynamics and physiological implications. These include blood, plasma, saliva, urine, cerebrospinal fluid (CSF), and hair. Specific types of biological material require various methodological approaches [21]. Cortisol levels in blood, plasma, and saliva are typically measured to obtain information on baseline (unstimulated) cortisol concentrations. Additionally, by collecting multiple samples, the natural rhythm of cortisol secretion and its increase in response to stressors can be determined [22]. Cortisol secretion follows a distinct diurnal pattern, with a notable feature known as the Cortisol Awakening Response (CAR) [23]. This response is characterized by a rapid increase in cortisol levels within the first 30–45 min after waking. In healthy individuals, this rise can range from 38% to 75%, with an average increase of about 50% [24]. The CAR is superimposed on the natural circadian rhythm of cortisol secretion, which typically peaks in the early morning hours before awakening and declines throughout the day. The CAR is thought to prepare the body for the anticipated demands of the day, potentially enhancing alertness and cognitive function [25]. Understanding the CAR is important for assessing the reactivity of the hypothalamic–pituitary–adrenal (HPA) axis and its role in stress responses and overall health. Disruptions in the CAR, such as blunted or hyperreactive responses, are considered indicative of HPA axis dysregulation and may be associated with various health conditions, including stress-related disorders [26,27,28].

The Newcastle–Ottawa Scale (NOS) assigns a score based on three key domains: Selection, which evaluates the adequacy of participant selection, including the representativeness of the study groups and non-response rates; Comparability, which assesses how well the study groups are comparable, considering factors such as confounding variables and matching strategies; and Exposure, which evaluates how the exposure of interest (e.g., cortisol measurement, stress response) is assessed and whether the method used is reliable and valid. Each domain is assigned a score, and studies are awarded a maximum of 4 points for Selection, 2 points for Comparability, and 3 points for Exposure, for a total possible score of 9 points. Studies with scores of 7 or higher are considered to have acceptable methodological quality.

#### 3.1.1. Suicidal Behaviors

A meta-analysis that included 30 studies on individuals with SBs (n = 1775) and a control group (healthy individuals, n = 696; individuals with other psychiatric diagnoses, n = 1465) showed that cortisol levels were generally higher in individuals with SBs compared to healthy controls (moderate to large effect size). However, when compared to psychiatric controls, individuals with SBs exhibited lower overall cortisol levels (large effect size). Furthermore, the associations were found to depend on the time of day at which the sample was collected. It is important to note that the meta-analysis aggregated studies measuring unstimulated cortisol levels in various types of biological material including serum, plasma, CSF, and saliva [54]. 

A recent study, not included in the previous meta-analysis [54], has provided similar evidence. Baseline plasma cortisol levels were significantly elevated in a group of adults (mean age = 32.88 years) following a suicide attempt (n = 56) compared to a healthy control group (n = 56). Furthermore, in the subgroup of individuals who had attempted suicide more than once, the difference was even more significant. However, no significant differences were observed when compared to a group of individuals with psychiatric disorders but without suicide attempts (n = 31) [29].

Interestingly, in a prospective study conducted in a large population of patients with depressive disorders (n = 1094) and healthy controls (n = 884), a significant correlation was found between elevated blood cortisol levels and the occurrence of suicide attempts in the following year [30]. This association was even more significant when cortisol levels were combined with other serum markers, such as total cholesterol and folate [31]. In another prospective study, a significantly lower CAR was identified in adults (mean age = 27.74 years, range = 18–63 years) following suicide attempts (n = 53) and suicidal ideation without attempts (n = 52), along with flatter cortisol slopes from wake peak to 12 h, compared to the control group (n = 49). Interestingly, a prospectively lower CAR was associated with increased suicidal ideation at 6 months, although this relationship was not observed at 12 months [32].

In a follow-up study, a lower CAR was also found to be associated with psychological risk factors for suicide, such as lower levels of stress resilience, higher levels of socially prescribed perfectionism, anxiety, and impulsivity [33]. Conversely, a comprehensive review involving 1749 participants (ages 18 to 65 years) did not demonstrate a significant correlation between the CAR or baseline salivary cortisol levels and the prevalence of suicidal ideation or suicide attempts [55]. Abnormal cortisol levels may also be dependent on the sleep-wake cycle. In the study involving healthy adolescents (n = 35) and adolescents with major depressive disorder (n = 42), blood samples were collected over a 24-h period at 20-min intervals to measure cortisol levels. Approximately 7 years later, a longitudinal follow-up interview was conducted. The results indicated that participants who had experienced at least one major depressive episode during the observation period and had attempted suicide showed significantly higher cortisol levels 4, 6, and 12 h before sleep onset. Conversely, this subgroup showed reduced cortisol levels 2–4 h after falling asleep [34]. In another study of adolescents, the association between trauma, suicide attempts/suicidal ideation, and morning cortisol levels was examined in a community sample of adolescents (n = 89, mean age = 15.3 years). No significant associations were found between blood cortisol levels and trauma exposure, suicide attempts, and suicidal ideation [35].

#### 3.1.2. NSSI

Data on resting cortisol levels in blood or saliva remain inconsistent. In one study, daily HPA axis activity was assessed using serial salivary cortisol measurements in adolescents with NSSI (n = 26) and healthy controls (n = 26). An elevated CAR was observed in the NSSI group, with no differences in the diurnal slope or baseline salivary cortisol levels [36]. Similarly, another study focused on thyroid function in adolescents (ages 12–17 years) with NSSI found no association between blood baseline cortisol levels in the NSSI group (n = 117) and the healthy control group (n = 41) [37]. In a further study, adolescents and young adults with major depressive disorder (MDD) were assessed for adverse childhood events and the presence of NSSI. The MDD with NSSI group (n = 46) showed higher levels of adverse childhood experiences and lower serum cortisol levels compared to the MDD without NSSI group (n = 80). A recent 2-year cohort study assessed the impact of adverse childhood experiences (ACEs) and HPA axis functioning on the longitudinal course of NSSI in a sample of adolescents engaged in NSSI (n = 51, mean age: 15.00 years, range 12–17 years). Unstimulated cortisol levels (CAR, DSL, circadian slope), hair cortisol concentrations, and ACEs were assessed. The frequency of NSSI was monitored at 6, 12, and 24 months. It was found that ACEs and HPA axis function did not show main effects but did demonstrate interactive effects in predicting NSSI frequency over time: adolescents with low ACEs and increased CAR or a flattened DSL showed a sharp decline in NSSI frequency during the first year, followed by a subsequent increase in NSSI frequency during the second year. No significant interaction effect for any parameter was found in predicting changes in NSSI frequency over time [38].

### 3.2. Hair Cortisol Concentration

The recently introduced analysis of hair cortisol concentration (HCC) in the HPA axis research offers several significant advantages for measuring chronic stress levels [56]. Unlike cortisol levels measured in blood or saliva, which can fluctuate significantly due to acute stressors and the time of day, HCC provides a stable, long-term index of cortisol levels. This stability is attributed to the gradual and continuous incorporation of cortisol into growing hair, thus reflecting a cumulative measure rather than moment-to-moment variations [57]. HCC has been shown to present overall validity, high test-retest reliability, and acute/situational stability [58]. The collection of hair samples is straightforward and non-invasive; typically, a small sample is cut close to the scalp, usually from the posterior vertex region. This method is painless, quick, and can be performed in various settings without special preparation, making it highly convenient for both participants and researchers [59]. HCC offers a retrospective view of cortisol secretion over several months. Given that hair grows at an average rate of about 1 cm per month, a 3 cm segment of hair taken closest to the scalp can represent cortisol levels over the preceding three months [60]. This retrospective nature allows researchers to assess cumulative stress exposure and correlate them with past events or conditions without the need for continuous monitoring [61]. Consequently, this method has been employed in research to link HCC with the occurrence of mental disorders.

A recent systematic review of 22 studies associating HCC with psychiatric mental disorders suggested that elevated HCC is found in patients with psychosis and PTSD. However, no significant differences were noted for depression or anxiety disorders [62]. To date, only a limited number of studies have examined associations between hair cortisol levels and SBs, as well as NSSI. A study found significantly lower HCCs in individuals (mean age = 23, range = 15–30) after a suicide attempt (n = 38) compared to a group with suicidal ideation but no prior history of attempts (n = 40) and healthy controls (n = 37) [39]. Conversely, in a study of suicide completers (n = 45), individuals with depression (n = 20), and a healthy control group (n = 12), significantly higher HCC was found in hair samples taken post-mortem from suicide completers compared to the control group, while HCC in depressed patients was found to be intermediate [40]. In the previously mentioned study [36] on adolescents with NSSI, no significant changes in HCC levels were observed between individuals with a history of NSSI and the control group. However, a subsequent study, which examined a sample consisting of 32 adolescents engaging in NSSI (n = 16, mean age = 15.8 years) and their siblings (n = 16, mean age = 15.6 years), found significantly higher HCC levels in those with NSSI [41]. Thus, the results concerning cortisol levels in hair remain ambiguous, indicating a need for more prospective studies to clarify these findings.

### 3.3. Stimulated Cortisol Levels

To evaluate the function of the HPA axis in response to controlled stress, several well-established protocols are employed. The Trier Social Stress Test (TSST), developed by Kirschbaum in 1993, is a standardized procedure that involves public speaking and arithmetic tasks performed in front of an evaluating audience, which triggers a moderate stress response [23]. Another widely used protocol for assessing the HPA axis response to physical or painful stressors is the Cold Pressor Test (CPT), which involves immersing the hand in icy water for a specified period [63]. The Maastricht Acute Stress Test (MAST) combines psychosocial stressors with the cold pressor test to elicit a stronger stress response [64].

The HPA axis response, similar to the CAR, is measured using the area under the curve with respect to increase (AUCi) and the area under the curve with respect to ground (AUCg). AUCg represents the total amount of cortisol secreted one hour after waking, while AUCi represents the dynamic response, indicating how much cortisol levels rise in response to the stressor compared to baseline levels, excluding the influence of the baseline level [65]. 

#### 3.3.1. Suicidal Behaviors

In a study of children of parents with mood disorders (n = 208, mean age = 23.3 years, range 16–38 years), it was found that offspring with history of suicide attempts show lower total cortisol production (AUCg) compared to offspring with suicide ideation but no history of suicide attempts, non-suicidal offspring, and healthy controls. However, no significant differences were noted between groups in cortisol reactivity to the TSST (AUCi) [42]. 

Similar results were obtained in another study of adults (mean age = 26.84 years, range 18–62 years), in which salivary cortisol levels were assessed in response to psychosocial stress induced by the Maastricht Acute Stress Test (MAST). The group of adults after a suicide attempt (n = 49) was compared to individuals with suicidal ideation but no history of suicide attempts (n = 55) and with healthy controls (n = 48). SBs were also prospectively assessed at 1 and 6 months. The suicide attempt group showed lower AUCg in response to the MAST compared to control participants. Those with only suicidal ideation had intermediate cortisol levels. No significant alterations in AUCi were observed. Furthermore, participants in the suicide attempt group who had lower cortisol levels in response to the MAST were significantly more likely to report higher levels of suicidal ideation one month later, but not 6 months later [43].

In a study on adults with mood disorders (mean age = 38.73 years) using a modified TSST procedure, no differences were found in AUCi or AUCg. However, it was shown that suicide attempters (n = 22) had lower baseline salivary cortisol levels compared to non-attempters (n = 47) [44]. To obtain more precise data, a search for subtypes among individuals after suicide attempts was initiated. In the study, it was shown that in the group of depressed individuals (n = 67, mean age = 39.26 years), the responsiveness to the TSST (AUCi) was significantly higher among participants after a suicide attempt, who presented low levels of depressive symptoms and high impulsivity compared to the control group [45] similarly, it was found that there were no differences between adults (mean age = 31.9 years) after suicide attempts (n = 35) and healthy participants (n = 37) in baseline cortisol levels, total cortisol output, or responsiveness to the TSST. However, significantly elevated baseline cortisol levels, AUCg after the task, and higher cortisol responsiveness of the HPA axis to the TSST (AUCi) were observed in the subgroup with high impulsivity and aggression compared to the subgroup with low impulsivity and aggression [46].

The HPA axis response to the TSST was examined in relation to the severity of suicidal intent among adult participants (n = 68, mean age = 33.07 years). It was shown that individuals with higher suicidal intent had lower total cortisol output (AUCg) and lower responsiveness to the TSST (AUCi) compared to the low-intent group [47].

Studies on the response to the Trier Social Stress Test (TSST) in children and adolescents produced somewhat different results; however, these studies focused primarily on the presence of suicidal ideation (SI) rather than suicide attempts. For example, the response to the TSST among children bereaved by the sudden death of a parent (n = 114 bereaved, n = 109 non-bereaved controls, mean age = 12.3 years) was examined [48]. Participants were observed over an average period of 7 years, with suicidal ideation severity assessed at several time points. The participants were divided into two groups based on SI trajectories. Higher stress reactivity (AUCi) was found in those in the higher SI trajectory group. No differences were observed in AUCg after the TSST. Additionally, higher baseline cortisol levels showed a small-to-moderate effect in predicting future suicidal ideation over 18.5 months following the TSST. The study had some limitations such as potential selection bias, different follow-up times, and lack of a healthy control group.

In contrast, a good quality study on adolescent females at risk for SBs (n = 138, mean age = 14.13 years) was conducted to assess risk factors for SBs (i.e., depressive symptoms, impulsivity, pubertal status, and peer stress) as well as the presence of suicidal ideation prospectively for 3 months after the study [49]. Three distinct patterns of cortisol stress response were identified: blunted, hyperreactive, and moderate. Females in the hyperreactive group were found to be more likely to report lifetime suicidal ideation at baseline and more likely to report suicidal ideation three months later; a similar trend was observed for the blunted response group. A continuation of this study, involving a larger group (n = 220, mean age = 14.69 years, range = 12–16 years), expanded these findings. Observations were conducted quarterly for 18 months. It was found that a blunted laboratory HPA axis response to stress prospectively predicted SBs, and periods of increased peer stress were shown to increase the risk of SBs, but only among those with blunted cortisol responses [50]. 

#### 3.3.2. NSSI

A recent meta-analysis which focused on the biological stress response in individuals with NSSI (n = 954, mean age = 19.81 years) and control subjects (n = 1122, mean age = 18.65 years) found that individuals with NSSI show significantly lower cortisol responses to stress compared to control subjects, as well as a slower return to baseline cortisol levels after a stressor. However, these alterations were characterized by a small effect size. The analysis included studies investigating psychosocial stressors (based on the TSST, MAST, etc.) [66]. Only one of included studies examined cortisol response to a painful stimulus (Cold Pressor Test) and its results were found to be significantly different from the results of other studies. Notably, NSSI involves painful stimuli. Therefore, assessments based on such stimuli are likely to more accurately reflect the HPA axis response to self-harm. In this study, a group of adolescent girls with NSSI (n = 30, mean age = 15.27 years) and a healthy control group (n = 30, mean age = 15.27 years) were subjected to cold stimulation. The increase in cortisol level was observed in both groups after the exposure to pain. Furthermore, the responsiveness of cortisol in the NSSI group was significantly greater in comparison with controls after adjustment for other characteristics (health-related behaviors) [51]. The results are in line with findings from another study conducted in a significantly larger group of adolescents (n = 164 NSSI, n = 45 healthy control, mean age = 14.8 years), who were exposed to painful thermal stimulation. Additionally, it was found that the effect of increased cortisol secretion following pain stimulation depended on the severity of NSSI (measured by the frequency of self-harm in the past 6 months) [52]. 

Interestingly, the effect of psychosocial stressors in individuals with NSSI is consistent with findings from meta-analyses on other mental disorders [67,68]. 

#### 3.3.3. Cortisol Levels in DST

The dexamethasone suppression test (DST) is a diagnostic procedure used to assess adrenal function by measuring changes in cortisol levels following the administration of dexamethasone, a synthetic glucocorticoid. This test is primarily employed to diagnose Cushing’s syndrome, a condition characterized by excessive cortisol production [69]. The procedure involves administering a small dose of dexamethasone, usually in the evening, and measuring cortisol levels the following morning. In individuals without abnormal cortisol production, cortisol levels should be suppressed. Failure to suppress is considered an indicator of dysfunction in the negative feedback mechanism of the HPA axis [70]. The advantage of the DST over social stress protocols lies in the use of a precisely defined, structured pharmacological stimulus. 

In a meta-analysis study, a total of 43 studies were focused on the association between DST non-suppression and SBs, specifically examining suicide attempts and suicide completion separately. The meta-analysis produced 37 effect size estimates for suicide attempts (n = 3733) and 11 effect size estimates for suicide completion (n = 1626). Key findings indicated that DST non-suppression was significantly associated with completed suicide (with a moderate-to-large effect size (OR = 2.10)). However, no overall association was found between DST status and suicide attempts across all patient samples. Moderator analysis revealed that DST non-suppression was associated with suicide attempts in patient samples including various psychopathology of mood disorders, psychosis, substance use, and personality disorders [71]. The correlation between the DST and NSSI remains much less understood. In the most recent study addressing this topic, the DST was administered to adolescent girls with a history of depression (n = 28, mean age = 15.7 years) or both depression and NSSI (n = 29, mean age = 16.3 years). The study found that lower cortisol levels after the DST were statistically significantly associated with suicidal ideation and self-inflicted injury [53].

## 4. Discussion

### 4.1. Summary of Findings

This review underscores the complexity of the relationship between cortisol levels and self-harm behaviors, including SBs and NSSI. While cortisol, a key marker of HPA axis function, exhibits altered patterns in individuals with SBs and NSSI, these changes are not consistently observed across studies. There appears to be a link between elevated basal blood cortisol levels and SBs, as demonstrated by both a meta-analysis of 30 studies [54] and a recent study of moderate quality [29]. Additionally, higher cortisol levels have been proposed as a predictor of future suicide attempts. However, no study has yet confirmed a clear cause-and-effect relationship. In contrast, studies investigating the relationship between cortisol and NSSI in adolescents have not shown differences in baseline cortisol levels. Furthermore, longitudinal studies have failed to establish a direct link between cortisol levels and the severity of future NSSI episodes. Two high-quality studies examining the association between hair cortisol concentration (HCC) and SBs reported divergent results [39,40]. Notably, the study by [40] had a small control group consisting exclusively of women, which may introduce gender bias. More studies are needed to clarify these discrepancies. The association between HCC and NSSI has been even less explored. A large, high-quality study did not find an association, while a more recent study observed increased HCC in adolescents with NSSI compared to healthy controls. However, the strength of this result is limited by the small sample size and the heterogeneity of the data. Studies examining cortisol levels following stimulation show inconsistent results. Two high-quality studies [42,43] found reduced total cortisol output without changes in cortisol reactivity, although the studies differed in the type of stress test used (TSST vs. MAST). A lower-quality study did not replicate these findings, potentially due to the use of a modified version of the TSST, which may have affected the results. Several factors may influence the observed differences in cortisol patterns. As previously discussed, co-occurring depression, the severity of depressive symptoms, levels of suicidal intent, and traits such as impulsivity and aggression may all play a role. Future studies should take these co-factors into account when investigating the relationship between cortisol and self-harm behaviors.

In adolescents, high-quality longitudinal research has shown that a blunted cortisol response to the TSST is associated with the occurrence of suicidal thoughts and SBs. However, it is important to note that both studies had gender biases, which may limit the generalizability of their findings. The nature of the stressor used in these studies may be another important factor in understanding the relationship between cortisol and NSSI. For example, scores from pain-based stress tests differed from those obtained with psychosocial stress tests. This could indicate that individuals with NSSI may use self-inflicted pain as a compensatory mechanism to stimulate a blunted HPA axis response to psychosocial stressors. Further research is needed to confirm this hypothesis. Despite a large meta-analysis, the dexamethasone suppression test (DST) did not detect a clear relationship between cortisol levels and either SBs or NSSI. This suggests that the DST may not be a reliable tool for studying the relationship between cortisol and SBs.

### 4.2. Methodological Limitations

Despite the comprehensive approach taken in this narrative review, several limitations must be acknowledged. First, the decision to conduct a narrative review, while this allowed for a broader synthesis, it also limited the ability to quantitatively compare the findings across studies, which could have provided a more precise estimate of the effect of cortisol on SBs and NSSI. Furthermore, the exclusion of studies published in languages other than English may have introduced a language bias. Another limitation of the review lies in the inclusion of both adolescent and adult populations. While this broadens the scope of findings, it introduces variability in terms of age-related factors that could influence the cortisol–SB/NSSI relationship, making it challenging to isolate patterns specific to adolescents. When comparing HPA axis functioning between adolescents and adults, several key differences must be considered. Adolescents typically exhibit higher baseline cortisol levels and increased HPA axis reactivity in response to stress. Additionally, they may have less efficient feedback regulation and slower recovery after stress compared to adults [72]. The study selection process, while rigorous, also faced challenges due to the reliance on abstracts and titles for initial screening. While two independent reviewers were involved to minimize bias, there is still a possibility of subjective interpretation or oversight during the screening and inclusion of studies. Lastly, due to the broad nature of the included studies, some findings may have been underpowered due to small sample sizes, and the lack of statistical meta-analysis meant that the review could not calculate pooled effect sizes, which would have provided more reliable estimates of the relationship between cortisol levels and suicidal behavior or NSSI.

### 4.3. Clinical Implications

Understanding the role of cortisol levels in individuals with SBs or NSSI holds the potential for important clinical applications in the future. However, at this stage, the relationship between cortisol levels and these behaviors remains complex and not yet fully understood. While research suggests that cortisol dysregulation may play a role in both SBs and NSSI, we currently lack conclusive evidence about the specific cortisol response patterns that characterize individuals with these behaviors. At present, understanding the potential connection between cortisol levels and SBs or NSSI may offer a valuable direction for future clinical practice. Once clearer patterns are established, cortisol levels could serve as a biomarker to help identify individuals at higher risk of engaging in SBs or NSSI. This could, in turn, guide the selection of targeted interventions, such as Cognitive Behavioral Therapy (CBT), Dialectical Behavior Therapy (DBT), or Collaborative Assessment and Management of Suicidality (CAMS), which have demonstrated efficacy in reducing suicidality and NSSI in clinical populations. Ideally, once research clarifies the cortisol–behavior link, clinicians could use this information to tailor these evidence-based therapies to better address the individual’s specific physiological and psychological stress responses. Moreover, the identification of cortisol dysregulation could pave the way for the development of therapeutic approaches aimed at regulating the HPA axis. For example, mindfulness-based interventions, exercise programs, and pharmacotherapies that target the normalization of cortisol levels might eventually prove beneficial in mitigating the dysregulated stress response often observed in individuals with suicidality or NSSI.

### 4.4. Future Research Directions

Future research on cortisol’s relationship to SBs and NSSI should prioritize a longitudinal design to track changes in cortisol levels over time, providing stronger causal inferences than cross-sectional studies. A larger, more diverse sample would enhance statistical power and generalizability, addressing the small sample sizes seen in some existing studies. It is crucial to include a broad range of participants, both with and without psychiatric conditions, and ensure representation from individuals with varying severities of NSSI and suicidal ideation/behavior, which would increase the applicability of findings. Furthermore, studies should consider gender as a separate factor or ensure equal representation of male and female participants to avoid gender bias. Regarding cortisol measurement, researchers should assess cortisol levels at multiple time points, including baseline, post-stress test, and follow-ups, to observe dynamic changes, such as cortisol reactivity and diurnal patterns, rather than relying on single-time point measures. Using a variety of cortisol biomaterials, such as saliva, hair, and blood, will allow comparisons of their predictive validity for suicidality and NSSI, offering complementary insights into long-term versus acute stress responses. Additionally, employing well-established stress induction methods like the TSST and incorporating tests like the CPT will help assess cortisol responses to stress and recovery phases, particularly regarding pain-related stress responses in NSSI. Emerging technologies such as electrochemical antibody-based sensors hold promise for real-time, continuous monitoring of cortisol levels through non-invasive, portable devices [73]. These sensors are cost-effective, comfortable, and sensitive, with the potential to enhance cortisol monitoring in both research and clinical settings [74,75]. Furthermore, studies should carefully control for key variables such as psychiatric diagnoses, hormonal status, medication use, and history of childhood trauma, as these factors may influence cortisol secretion and outcomes related to NSSI and SBs. A broad range of outcomes, including suicidal ideation, suicide attempts, and completed suicides, should be rigorously measured using validated scales, alongside a clear distinction between the frequency and severity of NSSI. Other psychological risk factors, such as impulsivity, aggression, and emotion regulation difficulties, should also be assessed for their interaction with cortisol responses. Methodological rigor is essential, with efforts to minimize task modifications to stress protocols to ensure consistency. Finally, findings should be replicated across diverse cohorts and settings to validate results and cross-validate with other stress biomarkers, such as inflammatory markers, to enhance generalizability.

## Figures and Tables

**Table 1:** Brief overview of studies included in a narrative review.

Authors [Ref No.]	Year	Mean Age (yrs)	N and Characteristics of Participants	Type of Study	Cortisol Measurement, Test	Main Results	Main Limitations	NOS Score	Selection	Comparability	Exposure
Genis-Mendoza et al. [29]	2022	32.88	Suicide attempt: 56, healthy controls: 56	Cross-sectional	Unstimulated cortisol (plasma)	Elevated baseline plasma cortisol levels in suicide attempt group compared to healthy controls.	Small sample size. Missing key factors: important variables like hormone use and therapy were not considered. Non-response data missing: no information on non-respondents, affecting reliability.	6/9	3/4	1/2	2/3
Choi et al. [30]	2022	range 17–85	Depressive disorder: 1094, healthy controls: 884	Longitudinal	Unstimulated cortisol (blood)	Elevated blood cortisol levels correlated with future suicide attempts.	Reliance on self-reported stress data.	8/9	3/4	2/2	3/3
Kim et al. [31]	2023	range 17–86	Depressive disorder: 1094, healthy controls: 884	Longitudinal	Unstimulated cortisol (blood)	Combined cortisol with other markers (such as total cholesterol and folate) predicted future suicide attempts.	Biomarker measurement at baseline only. Small numerical differences in biomarkers. Low number of suicide events. Lack of consideration for early antidepressant effects. Broad age range.	8/9	3/4	2/2	3/3
O'Connor et al. [32]	2020	27.74 (range 18–63)	Suicide attempts: 53, suicidal ideation: 52, control: 49	Longitudinal	Unstimulated cortisol (saliva)	Lower CAR and flatter cortisol slopes in suicide attempt group, association with increased suicidal ideation.	Small sample size. Retrospective self-report bias: the Childhood Trauma Questionnaire (CTQ) used for assessing childhood trauma relies on retrospective self-reports. Lack of clinical diagnoses: the study did not assess formal psychiatric diagnoses.	9/9	4/4	2/2	3/3
O'Connor et al. [33]	2021	27.74 (range 18–63)	Suicide attempts: 53, suicidal ideation: 52, control: 49	Longitudinal	Unstimulated cortisol (saliva)	Lower CAR associated with psychological risk factors for suicide.	Small sample size. Retrospective self-report bias: the Childhood Trauma Questionnaire (CTQ) used for assessing childhood trauma relies on retrospective self-reports. Lack of clinical diagnoses: the study did not assess formal psychiatric diagnoses.	9/9	4/4	2/2	3/3
Mathew et al. [34]	2003	first day: 15.00 years, follow up: ~7 years later	Healthy adolescents: 35, major depressive disorder adolescents: 42	Longitudinal	Unstimulated cortisol (blood)	Higher pre-sleep cortisol levels in MDD and suicide attempt group, lower levels of post-sleep cortisol.	High rate of control group conversion to depression. Small sample size. Potential retrospective recall bias.	8/9	3/4	2/2	3/3
Young [35]	2010	15.3	Adolescents: 501	Cross-sectional	Unstimulated cortisol (blood)	No association between cortisol and trauma, suicide attempts, or ideation.	Inconsistent findings and small effect sizes: Gender interactions. Trauma assessment limitations: lack of differentiation between recent and distant trauma. Non-representative sample. Single-time-point saliva cortisol assessments.	8/9	3/4	2/2	3/3
Reichl et al. [36]	2016	16.25 (range 14–18)	NSSI: 26, control: 26	Cross-sectional	Unstimulated cortisol (saliva), HCC	Elevated CAR in NSSI group, no differences in baseline cortisol, no differences in HCC.	Small sample size. Exclusion criteria: adolescents with acute suicidality, or other specific conditions were excluded. Missing data imputation. No long-term follow-up.	9/9	4/4	2/2	3/3
Flach et al. [37]	2021	range 12–17	NSSI adolescents: 117, control: 41	Cross-sectional	Unstimulated cortisol (blood)	No association between baseline cortisol levels and NSSI.	Gender bias: small proportion of male participants. Single-time-point cortisol assessments.	8/9	4/4	2/2	2/3
Reichl et al. [38]	2024	15.00 (range 12–17)	NSSI adolescents: 51	Longitudinal	Unstimulated cortisol (saliva), HCC	No main effect of ACEs or HPA axis on NSSI, interactive effect predicting NSSI frequency.	Small sample size. Inconsistent treatment across participants. Retrospective assessment of ACEs. Limited cortisol measurement: Cortisol was only measured on a few days (two or three) at limited time points. Missing data handling.	8/9	3/4	2/2	3/3
Melhem et al. [39]	2017	23 (range 15–30)	Suicide attempt: 38, suicidal ideation: 40, healthy controls: 37	Cross-sectional	HCC (hair)	Lower HCC in suicide attempt group compared to ideation and control groups.	Sample bias and representativeness: only a subset of the larger sample participated in the TSST. Exclusion criteria: adolescents with NSSI were excluded. Task modifications: the TSST was modified.	9/9	4/4	2/2	3/3
Karabatsiakis et al. [40]	2022	Not reported	Suicide completers: 45, depressed: 20, healthy controls: 12	Cross-sectional	HCC (hair)	Higher HCC in suicide completers compared to controls, intermediate in depressed group.	Gender bias: the control and MDD groups only included women, while the SC group included men and women. Single biomaterial focus: only hair cortisol level. Unmeasured hair characteristics: key factors like hair type, color, and treatment (except for visible bleaching) were not considered.	9/9	4/4	2/2	3/3
Reichl et al. [41]	2019	15.7	NSSI adolescents: 16, siblings: 15	Cross-sectional	HCC (hair)	Higher HCC in NSSI group compared to controls.	Small sample size. Heterogeneous sibling group: the sibling group varied in terms of birth order, gender, and psychopathology.	8/9	3/4	2/2	3/3
Melhem et al. [42]	2016	23.3 (range 16–38)	Offspring of parents with mood disorder, n= 208 (offspring with SA (n = 20), offspring with SRB (n = 20), NS offspring (n = 168), healthy controls, (n = 35))	Cross-sectional	AUCg and AUCi (saliva), TSST	Lower total cortisol output (AUCg) in offspring of suicide attempters, no differences in cortisol reactivity (AUCi) to the TSST.	Smal healthy control group. Exclusion criteria: adolescents with NSSI only were excluded. Task modifications: the TSST was modified.	7/9	3/4	2/2	2/3
O'Connor et al. [43]	2017	26.84 (range 18–62)	Suicide attempt: 49, suicidal ideation: 55, healthy controls: 48	Cross-sectional	AUCg and AUCi (saliva), MAST	Lower AUCg in response to the MAST in suicide attempt group, intermediate levels in suicidal ideation group, no AUCi differences.	Exclusion criteria: several participants were excluded for reasons like negative reactions to the stress test. Lack of clinical diagnoses for psychiatric disorders.	9/9	4/4	2/2	3/3
Keilp et al. [44]	2016	38.73	Mood disorders: 22 suicide attempters, 47 non-attempters	Cross-sectional	AUCi and AUCg (saliva), TSST	No differences in AUCi or AUCg; lower baseline cortisol in suicide attempters.	Sample size. Non-experimental control: the study's design was not entirely experimental, as it relied on a pilot social stress task and variations in procedural factors,	6/9	1/4	2/2	3/3
Alacreu-Crespo et al. [45]	2022	39.26	Depressed individuals: 67 (SA n= 41, without SA n = 26)	Cross-sectional	AUCi (saliva), TSST	Higher cortisol response to the TSST (AUCi) in suicide attempters with low depressive symptoms and high impulsivity.	Lack of healthy control group. Small sample size.	7/9	3/4	2/2	2/3
Stanley et al. [46]	2019	31.9	Suicide attempt: 35, healthy controls: 37	Cross-sectional	AUCg and AUCi (saliva), TSST	Elevated baseline cortisol, AUCg, and AUCi in subgroup with high impulsivity and aggression.	Lack of healthy control group, Small sample size.	6/9	2/4	2/2	2/3
Herzog et al. [47]	2023	33.07	Suicide attempt: 68	Cross-sectional	AUCg and AUCi (saliva), TSST	Lower AUCg and AUCi in high suicidal intent group compared to low intent.	Lack of healthy control group. Small sample size. Gender bias: small proportion of male participants.	8/9	4/4	2/2	2/3
Shalev et al. [48]	2019	12.3	Bereaved: 114, non-bereaved controls: 109	Cross-sectional	AUCi (saliva), TSST	Higher AUCi in those with more severe suicidal ideation.	Timing of the stressor task (TSST). Limited generalizability. The study focuses on offspring bereaved by sudden parental death. Potential selection bias.	6/9	2/4	2/2	2/3
Giletta et al. [49]	2015	14.13	Adolescent females at risk for SBs: 138	Longitudinal	AUCi (saliva), TSST	Hyperreactive and blunted group more likely to report suicidal ideation at baseline and 3 months later.	Focus on suicidal ideation only. Self-reported measures to assess predictors. Lack of control group. Gender bias: only female participants.	9/9	4/4	2/2	3/3
Eisenlohr-Moul et al. [50]	2018	14.69 (range 12–16)	Adolescent females at risk for SBs: 220	Longitudinal	AUCi (saliva), TSST	Blunted cortisol response to stress predicted future SBs, peer stress increased SB risk in blunted group.	Lack of healthy control group. Gender bias: only female participants.	7/9	3/4	1/2	3/3
Koenig et al. [51]	2017	15.27	NSSI adolescents: 30, control: 30	Cross-sectional	AUCi (saliva), Cold Pressor Test	Greater cortisol increase in NSSI group following painful cold pressor test compared to controls.	Lack of multiple cortisol samples. Comorbid disorders: n = 18 participants had comorbid BPD, but the sample size was too small to explore differences in pain responses between those with only NSSI and those with NSSI and BPD. Self-reported drug use.	9/9	4/4	2/2	3/3
van der Venne et al. [52]	2023	14.8	NSSI adolescents: 164, control: 45	Cross-sectional	AUCi (saliva), cold pressor test	Increased cortisol secretion after thermal pain in NSSI group, severity of NSSI affects cortisol response.	Sole use of NSSI frequency as an indicator. Differences between heat pain and actual NSSI methods. Gender bias: only female participants.	8/9	3/4	2/2	3/3
Beauchaine et al. [53]	2015	15.7 (depression), 16.3 (depression + NSSI)	Depression and NSSI: 28 (depression only), 29 (depression + NSSI)	Cross-sectional	DST (serum)	Lower cortisol levels after DST linked to suicidal ideation and self-inflicted injury.	No assessment of pubertal status. No measurement of pre-DST cortisol. Small sample size. Potential bias from blood draw reactions. Gender bias: only female participants.	6/9	3/4	1/2	2/3

NSSI, Non-suicidal self-injury; SB, Suicidal behavior; ACE, Adverse childhood experience; HCC, Hair cortisol concentration; CAR, Cortisol awakening response; TSST, Trier Social Stress Test; MAST, Maastricht Acute Stress Test; AUCi, Area under the curve with respect to increase; AUCg, Area under the curve with respect to ground; DST, Dexamethasone Suppression Test; SA, suicidial attempers; SRB, suicide-related behavior; NS, non-suicidial; BPD, borderline personality disorder.

## Supplementary Materials

The following supporting information can be downloaded at: https://www.mdpi.com/article/10.3390/brainsci15030287/s1. The supplementary materials file contains a description of the methodology along with the search strategy.

## Abbreviations

The following abbreviations are used in this manuscript:

NSSI	Non-suicidal self-injury
SB	Suicidal behavior
DST	Dexamethasone Suppression Test
HPA	Hypothalamic–pituitary–adrenal
ACE	Adverse childhood experience
HCC	Hair cortisol concentration
CAR	Cortisol awakening response
TSST	Trier Social Stress Test
MAST	Maastricht Acute Stress Test
CPT	Cold Pressor Test
AUCi	Area under the curve with respect to increase
AUCg	Area under the curve with respect to ground
