# Supplementary material for: Do Cortisol Levels Play a Role in Suicidal Behaviors and Non-Suicidal Self-Injuries in Children and Adolescents?—A Narrative Review
Source: Brain Sci. 2025 Mar 8;15(3):287. doi: 10.3390/brainsci15030287 (PMC11940228; doi:10.3390/brainsci15030287)
Supplement: Supplementary file 1 [file brainsci-15-00287-s001.zip › brainsci-3484856-supplementary.pdf]

Search strategy

Literature search strategy for narrative review: cortisol, suicidal behaviors (SBs), and non-suicidal self-Injury (NSSI) in children and adolescents.

1. Databases to Search:

PubMed;
Web of Science;
Google Scholar.

2. Search Terms: The search will combine the following terms using AND and OR operators:

Cortisol-related terms:
"cortisol";
"glucocorticoid";
"HPA axis";
Suicidal behaviors and non-suicidal self-injury-related terms:
"suicid*";
"non-suicidal self-injury";
"NSSI";
"self-injury";
"self-harm".
Age-related terms:
"adolescence";
"adolescent";
"teen*";
"underage";
"children".
Sample Search String:
("cortisol" or "glucocorticoid" or "HPA axis") and ("suicid*" or "non-suicidal self-injury" or "NSSI" or "self-injury" or "self-harm") and ("adolescence" or "adolescent" or "teen*" or "underage" or "children")

3. Search Filters:

Language: English (due to resource constraints);
Date: Articles published up to November 2024;
Exclusion of animal studies: focus will be exclusively on human studies;
Exclusion of irrelevant studies: exclude studies that do not report cortisol concentrations or do not focus on suicidality or NSSI.

4. Manual Search:

A manual search of the reference lists of included studies will be conducted to identify any relevant publications that may have been overlooked during the initial electronic search.

Study Selection and Screening Process

1. Removal of Duplicates:

Duplicate articles will be removed using reference management software (e.g., EndNote, Rayyan).

2. Title and Abstract Screening:

The first level of screening will be based on the titles and abstracts of articles. The aim is to determine whether the articles are relevant to the research question.
Screening will be performed independently by two authors, with disagreements resolved by discussion and consensus.

3. Full-Text Screening:

Full-text articles of potentially eligible studies will be retrieved for further screening.
Studies will be assessed for eligibility based on the inclusion and exclusion criteria.

    a. Inclusion Criteria:

Human studies examining cortisol concentrations in relation to suicidal behavior or NSSI in adolescents or adults;
Studies that include both male and female participants diagnosed with psychiatric disorders (excluding somatic diseases, neurological disorders, intellectual disabilities, and autism);
Only English language studies will be considered.

b. Exclusion Criteria:

Animal studies or studies that do not involve human subjects;
Studies that do not report cortisol levels or do not focus on suicidal behavior or NSSI;
Articles published in languages other than English.

4. Data Extraction and Synthesis:

For eligible studies, key information will be extracted including:
Study design: cohort, cross-sectional, longitudinal, etc.;
Sample characteristics: age, gender, psychiatric diagnoses;
Methods of cortisol assessment: blood, saliva, hair, etc.;
Findings: eelationship between cortisol levels and suicidality or NSSI;
Study limitations: noted limitations reported in the study.

Narrative Synthesis Approach

Given the methodological heterogeneity across the studies, a narrative synthesis will be employed. This approach allows the integration of diverse findings and provides a broader overview of the literature, identifying common themes, methodological trends, and differences across studies. The synthesis will be descriptive rather than statistical, as the varied designs and outcome measures across studies do not lend themselves to meta-analysis.
Key aspects of the synthesis will include:
Identification of patterns across studies that explore the relationship between cortisol levels and suicidal behaviors or NSSI;
Critical evaluation of study methods to address potential biases and methodological limitations;
Examination of differences in results, considering factors such as age, cortisol measurement methods, and study designs.

Risk of Bias and Quality Appraisal

To ensure the reliability of the findings, the methodological quality of the studies will be assessed using appropriate tools, such as the Newcastle–Ottawa Scale for cohort and case-control studies. This appraisal will help identify studies with potential biases and methodological flaws. Any studies with high risk of bias will be noted, and their impact on the overall synthesis will be discussed.
The appraisal will include the following:
Study design (e.g., cohort, cross-sectional);
Sampling methods and sample size;
Cortisol measurement methods (blood, saliva, hair);
Potential conflicts of interest.

Statistical Considerations

Due to the methodological diversity and inconsistent outcome measures across the included studies, statistical meta-analysis will not be conducted. Instead, a descriptive summary of findings will be provided, highlighting the relationships between cortisol levels and suicidal behaviors or NSSI. Effect sizes will be noted where possible, to illustrate the magnitude of differences observed between groups or conditions.
